# Inaugural Thesis for Graduation in the Atlanta Medical College

**Published:** 1881-04

**Authors:** D. W. Graham

**Affiliations:** McVille, Ga.


ATLANTA
### Medical and Surgical Journal.

VOL. XIX.] APRIL—1881. [NO. 1.

### Original Communications.

### INAUGURAL THESIS FOR GRADUATION IN THE ATLANTA MEDICAL COLLEGE, SESSION OF 1880-81, ON THE ACTION OF REMEDIES.

BY D. W. GRAHAM, McVILLE, GA.

We can conceive of no subject within the scope of the professional persuits of medical men more interesting than, and, we think, none so worthy of, thought, investigation as the one upon which we propose to write this essay.

For the rational and successful treatment of disease we must not only thoroughly understand the nature of the pathological conditions to which our remedies are directed, but as well we must have a correct knowledge of the physiological action of the remedies employed. With this knowledge we are prepared to accomplish all that can possibly be done in the treatment and cure of disease, and for the relief of human suffering. Without it we are totally unable to combat with any degree of success our common enemy—disease. In other words, we are empirics, practicing medicine after the routine of style, not according to medicine, that exhalted position it deserves to occupy as a science.

There is a fascinating tendency in the minds of many students to study remedies according to the diseases in which they are, or have been, used by others before us—administering certain remedies as specifics for the cure of particular diseases, to the exclusion of their physiological action. This is one of the greatest barriers to the successful study and progress of rational medicine.

The difference between the physiological action of a remedy and the therapeutic result of that action is world-wide. By the physiological action of a remedy is meant that which it exerted upon an organ or tissue in health, as well as in a state of disease. The therapeutic result of a remedy is the secondary effect produced by the remedy, and follows as the result of the physiological action, and is more or less permanent. This must be fairly understood by the practitioner for the rational treatment of disease.

Many (and we think extreme) theories have been advanced, by both the older and modern writers, to account for the action of remedies. We will give a few of these brief notice.

There ever has been a tendency in the human mind to explain everything, and it is but natural that our forefathers, who knew little of chemistry or physiology, should attempt to account for the action of all remedies, both local and elective, through mechanical process. With this idea, they claimed that the shapes of the minute particles of remedies are sufficient to account for their several actions.

Some writers attempt to explain the action of remedies through the similarity of the physical properties of the molecules composing the remedy, and those of particular organs or tissues of the body, and that the action of the remedy is exerted on a particular organ or tissue from the affinity thus existing.

Others attempt the explanation of the action of remedies by their sensible properties, and the botanical characteristics of the plants from which they are obtained. While these are theories plausible enough, we have not facts sufficient to sustain them.

The salts of the sulphate of zinc and sulphate of magnesia have quite similar sensible properties, while their physiological actions are very different. Again, the sensible properties of the sulphates of copper and zinc are very unlike, while their phyiological actions are almost identical.

The maxim of Hypocrates was "*contraria contrari curantur.*" With this idea we suppose cathartics alone would be given in constipation, no matter what the cause of the affection.

The rule of Hahnemann (the homœopathist) is "*similia similibus curau'er.*" From this we are to infer that his followers would administer opium in congestion of the brain, strychnia in *tetanus*, etc.

Many other theories have been promulgated to explain the action of remedies, but there is between those extremes a "happy medium," and we now know there are various ways in which remedies act in the cure of disease.

There are two grand divisions of remedies, viz : *Local and elective.* Remedies of the first division are said to act locally, as in the local application of a remedy for its effect upon the skin, or mucous membrane. We introduce astringents and demulcents into the stomach for their local impression on its mucous membrane. Hence we see local, as well as elective remedies, are given internally, but for very different objects. The one for its local impression, the other for absorption into the circulation, in order to get its action upon the organ or organs of its selection.

Elective remedies select particular organs or tissues of body, to the exclusion of others upon which to exert their action. Thus, opium selects the brain; oil of turpentine, the mucous membrane; and veratrum viride, the heart. It is said remedies of this division must enter the circulation for their impression. But we take the position that it is not necessary in every instance to have the remedy enter the circulation for its elective action; and that some remedies have both local and elective action. Belladonna is an elective remedy when administered internally and has the effect of dilating the pupils of both eyes. In this case it first enters the circulation. Again, if we apply a solution of atropia—the active principle of belladonna—to the eye locally, it has the elective power of dilating the pupil, and does so without entering the circulation. This is proven by the fact that the action is had upon the eye upon which the remedy is applied, without any influence whatever being exerted upon the eye of the opposite side in the same subject. If the remedy had first to enter the circulation for its action both eyes would evidently be equally impressed. This we deem sufficient evidence to sustain our position.

These were formerly termed constitutional or general remedies, and were supposed to act in a general manner upon the whole body. This opinion we now know to be erroneous. These remedies may, by their action on the organs or tissues of their selection, modify to a certain extent, all the organs of the body, but only as a result of their physiological action as a vital controlling organ.

Remedies are said to act through three distinct processes, viz: mechanical, chemical and vital. We obtain the mechanical action by merely placing the remedy in contact with the surface of the body; and it is intended either to protect or irritate the part, as the application of olive or linseed oil to a denuded surface to protect it from external irritating causes, and the administration of pulverized charcoal for its irritating effect on the mucous membrane of the intestines. Hence, it will be seen, these remedies are employed both externally and internally, and in either case to protect or irritate.

Remedies which act through chemical process make their impression on adventitious substances to the extent of their destruction; and also modify the composition of the blood and other fluids of the body. In this class are found both local and elective remedies. We have a good example of the first in anthelmentics and escharotics; of the second in hæmatinics, spanæmics and catalytic agents. The actions of these remedies are accounted for through purely chemical principles.

There is a third class of remedies whose action can be accounted for neither on mechanical or chemical principles. By their action modifications are wrought in the capillary circulation, and vitality of a part; and for this reason it is said they act through vital processes. We must confess, the exact manner of this action seems to us to be but little understood. There seems to be a striking analogy between the explanation given of this process and the diagnosis made by some doctors, when they have a train of not very well defined nervous symptoms, and for want of a name they say the patient is suffering from liver disease. But upon this hypothesis, probably, is based the most plausible explanation of the action of a large proportion of remedies.

To sum up the whole matter, we may say there are too grand divisions of remedies, *local and elective*, and that there is but one natural specific action of a remedy, and that action is physiological, and may be had through mechanical, chemical or vital process.

Most remedies (not all) have two distinct actions, *primary and secondary;* at least, these two actions have been claimed by most writers on the subject.

Primary action is the first impression made on an organ or tissue by a remedy. This is often very transient, and is followed by the secondary, and usually, the more important action of the remedy. The primary effect of alcohol is that of unusual stimulation and excitement to the brain. This lasts but a short time, and gives place to the secondary, which is the more permanent action.

Local as well as elective remedies have these two actions. Thus, in the application of a cauterant or catheretic, the primary action is that of excitement, while the secondary is quite the opposite, leaving the tissue with less vitality than before the application. We have some doubt as to whether there can be two distinct actions exerted upon the same organ by the same dose of one remedy. We think what has been called the secondary action is but the therapeutic result of the primary impression of the remedy.

The therapeutic results of these actions are either direct or indirect when the curative change is produced in the part upon which the action of the remedy is exerted; indirect when the change is wrought upon a diseased organ by the action of the remedy upon another organ in a state of health.

Many circumstances modify the action of remedies, some to a greater, some to a less extent. For the rational practice of medicine it is all important that the practitioner should thoroughly understand and take into due consideration these circumstances, as serious results may come from the administration of medicines without refer ence to the surroundings or peculiar susceptibility of the patient to the action of certain remedies. We shall briefly speak of a few of the more important of these circumstances.

*Idiosyncrasy.*—This is a peculiar conformation of the constitution which prevents the remedy from exerting its known physiological action upon the system. We should always give due credit to the statement of the patient in regard to the action of certain remedies in his particular case. It is known that an ordinary dose of morphia, in certain persons, will bring on spasm of the colon or colic, while at the same time we know this is not the usual action of this remedy.

*Sex.*—Usually but little or no difference need be observed in the dose of medicine given the male or female; yet, as woman is more susceptible to mental influences and nervous impressions than man, we should administer neurotics to the female with greater caution. The functions of the uterus modify the action of remedies. No sensible physician would administer drastic cathartics or mercury to an ordinary extent to a pregnant female, in the treatment of diseases which, under different circumstances, would indicate the use of these remedies.

*Temperament.*—Four in number: the bilious, lymphatic, nervous and sanguine. These must be taken into consideration in order to calculate the degree of physiological action to be had by a certain remedy, and the therapeutic changes produced by such action. Exciting neurotics should be given with caution in the nervous temperament, as persons in whom the nervous influences predominate are peculiarly susceptible to the impressions of this class of remedies. Depletion and the active antiphlogistic treatment may be carried to almost any extent desired in the sanguine temperament with good effect, while such a course is not at all admissible in the lymphatic temperament, and if attempted and persevered in would soon result in the destruction of the patient.

*Climate.*—The inhabitants of warm climates are more susceptible to the impression of opiates than those residing in cold climates. The same may be said of calorifics

—heat-producing agents—a much larger quantity being necessary to keep up the required temperature of the body in the North than in the South. On the other hand, remedies intended to excite the liver are required in much larger doses in Southern than Northern climates.

*Age* modifies the action of remedies to a very great extent. A given medicine is required in less quantity for a given action in the aged than would be required in the same person during the prime of life. This fact should receive due consideration in the administration of medicine to the aged. Again, it is known that in children opiates are required in less quantity for a given impression, in proportion to age, than in the adult; while in the administration of mercury the opposite is the rule. For some cause, not very well understood, children are, in proportion to age, much less susceptible to the impression of this remedy than the adult. A dose of mercury, which would produce ptyalism in the adult, would not do so in a child even of a few years old.

With an honest conviction of having given a candid and impartial description of the true physiological action of remedies, with the lights before us, we here close our remarks.

